# Pterostilbene, a Resveratrol Derivative, Improves Ovary Function by Upregulating Antioxidant Defenses in the Aging Chickens via Increased SIRT1/Nrf2 Expression

**DOI:** 10.3390/antiox13080935

**Published:** 2024-08-01

**Authors:** Xinyu Wang, Qiongyu Yuan, Yingyu Xiao, Xiangyu Cai, Zhaoyu Yang, Weidong Zeng, Yuling Mi, Caiqiao Zhang

**Affiliations:** Department of Veterinary Medicine, College of Animal Sciences, Zhejiang University, Hangzhou 310058, China; 22217098@zju.edu.cn (X.W.); qiongyu@zju.edu.cn (Q.Y.); 22317125@zju.edu.cn (Y.X.); 22317105@zju.edu.cn (X.C.); 12117050@zju.edu.cn (Z.Y.); zengwd@zju.edu.cn (W.Z.)

**Keywords:** Pterostilbene, oxidative stress, apoptosis, ovarian aging, chicken

## Abstract

Oxidative stress is recognized as a prominent factor contributing to follicular atresia and ovarian aging, which leads to decreased laying performance in hens. Reducing oxidative stress can improve ovarian function and prolong the laying period in poultry. This study investigates the impact of Pterostilbene (PTS), a natural antioxidant, on ovarian oxidative stress in low-laying chickens. Thirty-six Hy-Line White laying chickens were evenly divided into four groups and fed diets containing varying doses of PTS for 15 consecutive days. The results showed that dietary supplementation with PTS significantly increased the laying rate, with the most effective group exhibiting a remarkable 42.7% increase. Furthermore, PTS significantly enhanced the antioxidant capacity of aging laying hens, as evidenced by increased levels of glutathione, glutathione peroxidase, superoxide dismutase, catalase, and total antioxidant capacity in the ovaries, livers, and serum. Subsequent experiments revealed decreased expressions of Bax, Caspase-3, and γ-H2AX, along with an increased expression of BCL-2 in the ovaries and livers of laying hens. PTS supplementation also positively affects fat metabolism by reducing abdominal fat accumulation and promoting fat transfer from the liver to the ovary. To elucidate the mechanism underlying the effects of PTS on ovarian function, a series of in vitro experiments were conducted. These in vitro experiments revealed that PTS pretreatment restored the antioxidant capacity of D-galactose-induced small white follicles by upregulating SIRT1/Nrf2 expression. This protective effect was inhibited by EX-527, a specific inhibitor of SIRT1. These findings suggest that the natural antioxidant PTS has the potential to regulate cell apoptosis and fat metabolism in laying chickens by ameliorating oxidative stress, thereby enhancing laying performance.

## 1. Introduction

The follicle is composed of oocytes, granulosa cells (GCs), and theca cells (TCs), serving as the basic functional unit of the female reproductive system [1]. Alterations in the microenvironment of follicle cells, such as oxidative stress (OS) and excessive accumulation of reactive oxygen species (ROS), can lead to nuclear DNA damage, mitochondrial dysfunction, cell apoptosis, and increased incidence of inflammation [2]. These changes can adversely affect ovarian health and accelerate the aging process of the ovary [3].

The study of laying chickens is of significant agricultural importance, with the ovary serving as a classic model for studying folliculogenesis. In chickens, the ovaries contain numerous follicles of varying sizes, categorized into pre-hierarchical and hierarchical follicles based on their diameter [4]. Pre-hierarchical follicles (PHFs) include small white follicles (<4 mm; SWFs), large white follicles (4–6 mm; LWFs), small yellow follicles (6–8 mm; SYFs), and large yellow follicles (≥9 mm; LYFs). Commercial egg production in laying chickens notably declines around 580 days of age, resulting in a significant loss for the poultry industry [5]. Under physiological conditions, follicular atresia occurs exclusively in pre-hierarchical follicles [6,7], which is why we usually selected SWFs at the early stage of follicle development as experimental subjects. Several studies have identified oxidative stress and apoptosis, induced by the accumulation of ROS in the follicles of aged laying chickens, as primary contributors to follicular atresia [8,9]. Furthermore, oxidative imbalance plays a significant role in the progression of liver diseases and associated metabolic disorders [10]. Additionally, aging is associated with an increase in body fat accumulation [11]. Hence, exploring antioxidative stress measures is crucial to improving the production performance of laying hens.

Under normal situations, the utilization of antioxidant enzymes and bio-antioxidants facilitates the dynamic clearance and equilibrium of intracellular ROS [12]. However, as organisms age, the ovarian antioxidant system’s ability to neutralize ROS is significantly diminished [13]. Sirtuin-1 (SIRT1), a member of the silent transcription regulator family, is implicated in lifespan regulation and aging delay and is considered a potential marker of decreased ovarian potential [14,15]. Meanwhile, the Nrf2 (NF-E2-related factor-2) transcription factor regulates antioxidant gene expression, induce various cellular defense mechanisms against oxidative stress and plays a crucial role in ovarian aging [16]. SIRT1 not only upregulates Nrf2 expression but also hinders Nrf2’s binding with Keap1/Cul3, thereby bolstering cellular antioxidant capacity [17]. Furthermore, SIRT1 has been shown to mitigate ovarian cells senescence in short-lived fish (*N. guentheri*) and decrease β-SA-gal expression in cells by activating Nrf2 [18].

Natural products with potential anti-ovarian aging effects, through modulation of various related pathways, are currently a focus of research. Many well-known natural plant extracts have a strong positive effect on reducing ovarian oxidative stress and regulating ovarian function [5,9,19,20]. For instance, Pterostilbene (PTS) is a phenolic substance extracted from grapes, berries, rosewood heart, and other plants. Numerous studies have demonstrated its antioxidant, anti-aging and anti-inflammatory effects [21,22,23,24]. PTS derived from resveratrol, replaces two hydroxyl structures with two methyl structures, thus enhancing its lipid solubility and bioavailability [25]. Several studies have demonstrated that PTS exhibits superior antioxidative stress and anti-apoptotic effects compared with resveratrol [26]. Furthermore, PTS has been shown to alleviate oxidative stress and ferroptosis in human GCs through the Nrf2/HO-1 pathway [27]. However, additional research is necessary to ascertain whether PTS can improve ovarian oxidative stress and enhance production performance in aged laying chickens.

As a reducing sugar, D-galactose (D-gal) generates advanced glycation end products (AGEs) during oxidative metabolism. The D-gal-induced ovarian aging model closely resembles the natural aging process in animals, making it widely employed in constructing aging ovarian models [28]. Compared to the natural aging model, the D-gal-induced accelerated aging model is most commonly used due to its shorter duration, fewer side effects, lower cost, easier application, higher survival rate, and greater convenience [29]. Therefore, in this experiment, D-gal was chosen to establish an in vitro aging model to investigate the protective effects of PTS on SWFs.

In this study, we investigated the protective effect of PTS against oxidative stress injury in the chicken ovaries. Subsequently, PTS was administered directly to naturally aging laying chickens to observe its potential in alleviating oxidative stress damage to the ovaries under normal physiological conditions. Our findings may provide new insights into the mechanisms through which PTS is involved in anti-ovarian aging.

## 2. Materials and Methods

### 2.1. Animals

Hy-Line White laying chickens were acquired from a nearby commercial poultry farm. The chickens had unlimited access to food and water and were exposed to a regulated light schedule of 14 h of illumination, followed by 10 h of darkness. Ambient humidity and temperature were maintained at approximately 60% and 38.5 °C, respectively [30]. The composition of the basic feed given to laying hens remained consistent with that previously published by the laboratory [31]. Every protocol adhered to the Zhejiang University (ZJU 20220085) Guidelines for Laboratory Animal Care and Use.

For the in vitro experiment, SWFs were collected from laying chickens approximately around D280, with a variation of up to 10 days. Each trial included at least six hens.

For the in vivo experiment, following relevant literature and data [32,33]. Thirty-six D580 hens were randomly and evenly divided into four distinct groups (Control, high-, medium-, and low-dosage). Each group had three replicates, with each replicate containing three hens housed in flat cages measuring 70 × 50 × 64 cm. For 15 straight days, hens were given a basic diet enhanced with 100, 200, and 400 mg/kg of PTS. At the end of the experiment, three hens from each replicate group, were randomly chosen, weighed, and then slaughtered. Samples retained include ovaries, liver, and serum. Daily egg production rates were recorded. On the 15th day, we randomly selected and removed 6 eggs from each group to assess their quality parameters.

### 2.2. Plasma Analysis

Before euthanizing the hens, blood was drawn from their wing veins to prepare plasma. The plasma was then separated and stored at −20 °C. A biochemical autoanalyzer (Bs-300 Mindray, Shenzhen, China) was used to measure the concentrations of aspartate aminotransferase (AST), alanine aminotransferase (ALT), glucose (GLU), low-density lipoprotein (LDL), high-density lipoprotein (HDL), triglyceride (TG), and total cholesterol (TC).

### 2.3. Biochemical Analysis

Antioxidant capacity was assessed by measuring various indicators in liver, ovary, and serum according to the respective instructions. Specifically, malonaldehyde (MDA), hydrogen peroxide (H_2_O_2_), glutathione (GSH), glutathione peroxidase (GSH-Px), total superoxide dismutase (T-SOD), total antioxidant capacity (T-AOC), pyruvic acid and catalase (CAT) were quantified using kits from Nanjing Jiancheng Institute of Bioengineering, Nanjing, China.

### 2.4. Sample Collection

Ovaries and livers were isolated from laying hens and placed in sterile pre-cooled PBS, then washed three more times with pre-cooled PBS. The tissues were divided into small pieces using forceps and scissors. Subsequently, the tissue pieces were placed into 4% paraformaldehyde (BL539A, Biosharp, Shanghai, China) and OCT embedding agent (6502, Thermo Scientific, Waltham, MA, USA) for subsequent experiments. A part of the tissue was stored in a cryostorage tube and submerged in liquid nitrogen for preservation.

### 2.5. SWFs Culture and Treatment of Chemicals

SWFs were isolated from the ovary and subsequently rinsed with chilled, sterile PBS to eliminate any blood clots. After that SWFs were transferred to 48-well culture plates (Corning Inc., Corning, NY, USA). High-glucose DMEM (Hyclone, Tauranga, New Zealand) with 5% fetal bovine serum (FCS; Hyclone, UT, USA) was added and incubated at 38.5 °C with 5% CO_2_ for 72 h, with the medium being refreshed every 24 h.

To trigger senescence, a procedure using D-gal treatment was utilized. Briefly, SWFs separated from D280 hens were treat with different concentration of D-gal. Based on the evaluation of cell proliferation and apoptosis rates, and in comparison, with naturally aged laying hens, a dose of 200 mM D-gal for 48 h was chosen as optimal concentration for subsequent experiments.

Pterostilbene (purity ≥ 97%, CAS537-42-8, Yuanye, Shanghai, China) was initially dissolved in DMSO, and then diluted with DMEM. The final DMSO concentration in the medium remained below 0.1%. In a similar manner, SWFs were exposed to PTS at varying concentrations between 0.05 and 50 μM for a duration of 24 h. After evaluating the cell proliferation rates, 0.5 μM PTS was determined to be the deal concentration for future tests.

After treatment, the SWFs were collected and allocated for morphological observation and fluorescence immunohistochemistry after fixation in 4% paraformaldehyde, while the remaining samples were allocated for biochemical analysis and qRT-PCR.

#### 2.5.1. Experiment 1 Impact of PTS on D-Gal Triggered Aging in SWFs

SWFs from D280 chickens were randomly divided into 4 groups: control, 200 mM D-gal, 0.5 μM PTS +200 mM D-gal, and 0.5 μM PTS. Prior to the 48 h D-gal (200 mM) treatment, the cultured SWFs were treated with 0.5 μM PTS for 24 h. The total treatment duration was 72 h.

#### 2.5.2. Experiment 2 Treatment with Inhibitors

Before treatment with PTS (0.5 μM) and D-gal (200 mM), cultured SWFs underwent a 24 h pretreatment with 20 μM EX-527, a sirtuin1 (SIRT1) inhibitor (49843-98-3, MedChemExpress, Monmouth Junction, NJ, USA). EX-527 was dissolved first in DMSO and subsequently diluted with DMEM. Throughout the experiments, the DMSO content in the medium remained below 0.1%.

### 2.6. Morphological Observation

Tissues were rinsed over night with running water after fixation, dehydrated in graded ethanol, and then immersed in paraffin (60 °C) for 40 min before embedded. Sections of paraffin, each 5 μm thick, were prepared for subsequent experiments.

The samples were encased in an OCT medium (6502, Thermo Scientific, Waltham, MA, USA) and rapidly frozen using liquid nitrogen. The frozen samples were sliced into 10 μm thick sections with a CryoStar NX50 cryostat microtome (Thermo Scientific, Waltham, MA, USA).

#### 2.6.1. Hematoxylin and Eosin (H&E) Staining

Tissue sections were stained following standard protocols [34]. Subsequently, the sections were examined using an Eclipse 80i microscope. (Nikon, Tokyo, Japan).

#### 2.6.2. Oil Red O Staining

The cryosections were treated with Oil Red O dye (G1015-100ML; Servicebio, Wuhan, China) for a duration of 15 min at ambient temperature. Afterwards, the nuclei were stained with hematoxylin, and the samples were then visualized using an Eclipse 80i microscope (Nikon, Tokyo, Japan).

#### 2.6.3. TUNEL Staining

Deparaffinized paraffin sections were treated with the terminal deoxynucleotidyl transferase-mediated deoxyuridine triphosphate nick-end labeling (TUNEL) Bright Green Apoptosis Detection Kit (A11203, Vazyme, Nanjing, China), according to the manufacturer’s instructions. After the incubation period, images of the slides were examined using an IX70 fluorescence microscope (Olympus IX70, Tokyo, Japan).

#### 2.6.4. BrdU Staining

For the BrdU incorporation assay, SWFs were cultured with 10 μg/mL BrdU for 24 h. After treatment, SWFs were washed more than three times in pre-cooled PBS and then collected for subsequent determinations. Immunofluorescence (IF) staining was performed, as previously reported [23]. Subsequently, fluorescence images were observed using an IX70 fluorescence microscope (Olympus IX70, Tokyo, Japan).

### 2.7. RNA Extraction and qRT-PCR

The tissues were rinsed with pre-cooled PBS and then homogenized using Trizol (Invitrogen, Carlsbad, CA, USA). Afterward, RNA was isolated using conventional techniques, and 1 µg of this RNA was converted into cDNA using the HiScriptII 1st Strand cDNA Synthesis Kit (Vazyme). The expression levels of various genes were measured using real-time quantitative fluorescence PCR (qRT-PCR). The primer sequences used for qPCR analysis are detailed in Table 1. To standardize, all samples were normalized using the average expression of β-actin, employing the comparative cycle threshold method (2^−△△Ct^).

### 2.8. Analysis Using Western Blot Technique

After various treatments, different tissues were homogenized with RIPA Buffer (Fdbio, Hangzhou, China) supplemented with 1% PMSF (Meilunbio, Dalian, China) and 1% Protein Phosphatase Inhibitor (Solarbio, Beijing, China). Subsequently, centrifugation was performed to obtain the supernatant. The total protein concentrations were assessed using a BCA protein assay kit (Nanjing Jiancheng Institute of Bioengineering, Nanjing, China). A total of 30 μg of total protein was combined with SDS 5× loading buffer and RIPA to make a final volume of 10 μL. The mixture was then heated at 95 °C for 15 min. Subsequently, this mixture was loaded onto an SDS-PAGE gel, electrophoresed, and transferred to a 0.22 μm polyvinylidene difluoride (PVDF) membrane (Millipore, Bedford, MA, USA).

The PVDF membrane was blocked with 5% skim milk at room temperature for 2 h, followed by overnight incubation at 4 °C with different antibodies. These antibodies included mouse anti-SIRT1 (7-C5-B2, Huabio, Hangzhou, China); rabbit anti-Nrf2 (R1312-8, HuaBio, Hangzhou, China); rabbit anti-phospho-Nrf2 (SU 0334, HuaBio, Hangzhou, China), rabbit anti-CDK2 (ET 1602-6, HuaBio, Hangzhou, China); anti-PCNA (ab 29, Abcam, Cam-bridge, UK); rabbit anti-Bax (ER0907, Huabio, Hangzhou, China); rabbit anti-BCL-2 (ET 1610-20, Huabio, Hangzhou, China); rabbit anti-Caspase-3 (A 25309, Abclonal, Wuhan, China); rabbit anti-γ-H2AX (ET1602-2, HuaBio, Hangzhou, China); and anti-β-actin (AC 026, Abclonal, Wuhan, China).

The next day, the PVDF membrane was left at room temperature for 30–60 min, then incubated for another hour with the secondary antibody. Subsequently, the blots underwent three washes in TBST and were detected with the ECL Substrate Kit (FD 8030, Fdbio, Hangzhou, China). The levels of protein expression were measured with the help of ImageJ version 2.3.0 software. The expression of p-Nrf2 was normalized to total Nrf2, and p-ACC expression was normalized to total ACC, while all other protein expressions were normalized to β-actin.

### 2.9. Statistical Analysis

Each experiment was independently conducted at least 3 times, and the results were presented as the mean standard error of the mean (SEM). GraphPad Prism 9.0 software was used for statistical analysis and compared differences among groups using one-way ANOVA with Tukey’s multiple comparisons test or Student’s *t*-test. Statistical significance was defined as *p* < 0.05.

## 3. Results

### 3.1. Effects of PTS Supplementation on Laying Performance and Serum in Aged Hens

#### 3.1.1. Effects of PTS Supplementation on Egg Production and Follicle Development

**Figure 1:**
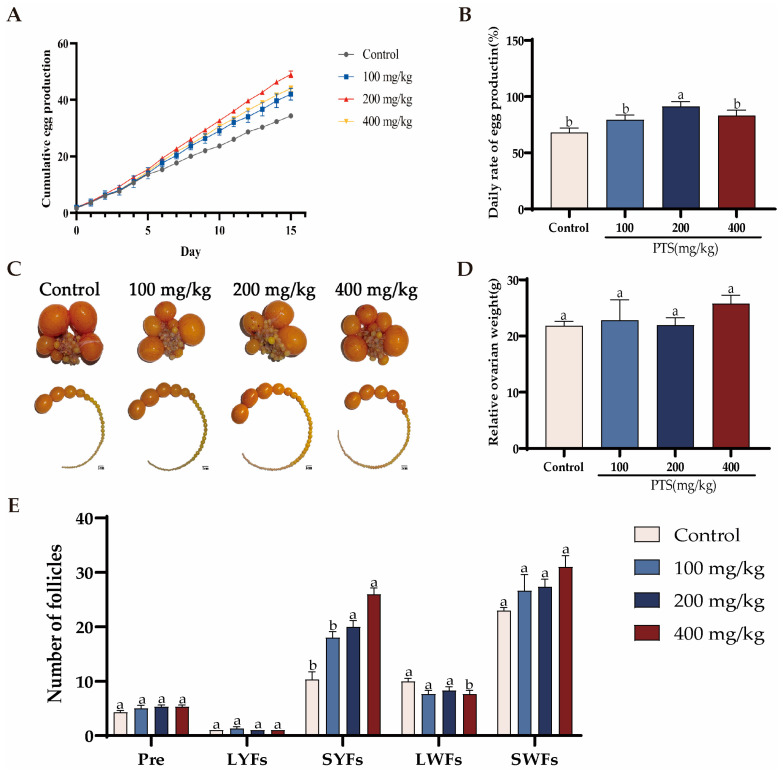
Effect of PTS supplementation on the laying performance of D580 hens. (**A**) Cumulative egg production over a span of 15 days measured in four distinct groups of laying hens. (**B**) Daily egg production rate of the laying hens recorded for each group over a period of 15 days. (**C**, **E**) Count of follicles at different grades in each of the four groups. Scale bar: 1 cm. (**D**) Changes of relative ovary weight was determined by calculating the ovary weight to body weight ratio. Significant differences between groups are shown by distinct lowercase letters in a test (*p* < 0.05).

To explore the effect of PTS on low egg-laying rate hens, 36 laying hens were randomly divided into four groups. Each group receiving a basal diet supplemented with different doses of PTS (0 mg/kg, 100 mg/kg, 200 mg/kg, 400 mg/kg) for 15 consecutive days. The effects of dietary supplementation with PTS on laying rate, follicle number, and ovary weight are shown in Figure 1. Daily cumulative egg production increased with the addition of PTS (Figure 1A). The highest daily laying rate was recorded in the 200 mg/kg dose group, representing a 42.7% increase compared to the control group (Figure 1B). Further analysis of PHFs showed that the number of SWFs and SYFs in the PTS fed groups exhibited a rising trend, but only the number of SYFs in the 200 mg/kg dose group and the 400 mg/kg dose group increased significantly compared to the control group (Figure 1C, E). However, there was no significant difference in ovary weight change between these four groups (Figure 1D).

Table 2 presents the physical quality of the eggs. The egg yolk color index improved in all three PTS supplement groups, while the Haugh Unit showed a significant change only in the 400 mg/kg group. However, there were no differences in egg weight, eggshell strength, and eggshell thickness between the groups fed PTS and the control group. (*p* > 0.05). 

#### 3.1.2. Effects of PTS Supplementation on Serum Antioxidant Capacity and Biochemical Parameters

Table 3 presents the biochemical parameters of each group of laying hens. The data indicate that PTS supplementation has no effect on serum ALT levels, and there was only a significant decrease in AST levels when the PTS content in the forage reaches 400 mg/kg, suggesting that feeding PTS does not induce liver damage. Moreover, the serum levels of TG and TC significantly decreased after feeding PTS, suggesting a potential impact on fat metabolism in laying hens. The content of GLU and HDL in serum was not significantly changed after feeding PTS.

**Figure 2:**
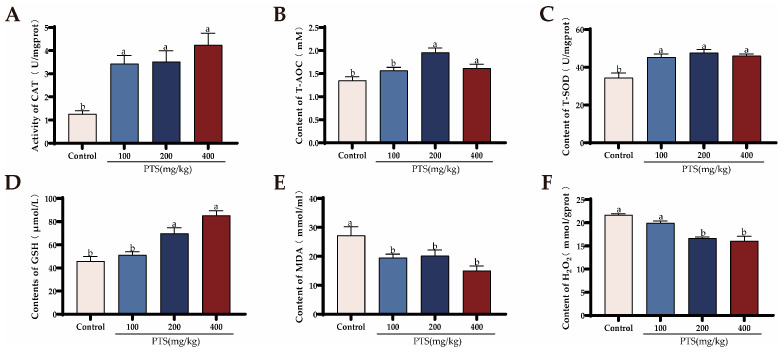
Effects of PTS supplementation on serum antioxidant capacity of laying hens. (**A**–**F**) Changes of serum antioxidant capacity of laying hens after PTS supplementation (CAT (**A**), T-AOC (**B**), SOD (**C**), GSH (**D**), MDA (**E**) and H_2_O_2_ (**F**)) content in serum. Significant differences between groups are shown by distinct lowercase letters in a test (*p* < 0.05).

Similarly, the serum antioxidant capacity assessment, revealed that PTS supplementation increased the levels of CAT (Figure 2A), and T-SOD (Figure 2C), while reducing the concentrations of MDA in the serum (Figure 2E). However, only when the PTS concentration reached 200 mg/kg and 400 mg/kg, were the serum T-AOC (Figure 2B) and GSH (Figure 2D) contents increased significantly, while the serum H_2_O_2_ (Figure 2F) content decreased.

### 3.2. PTS Supplementation Demonstrates Antioxidative Effects on Ovary and Liver In Vivo

**Figure 3:**
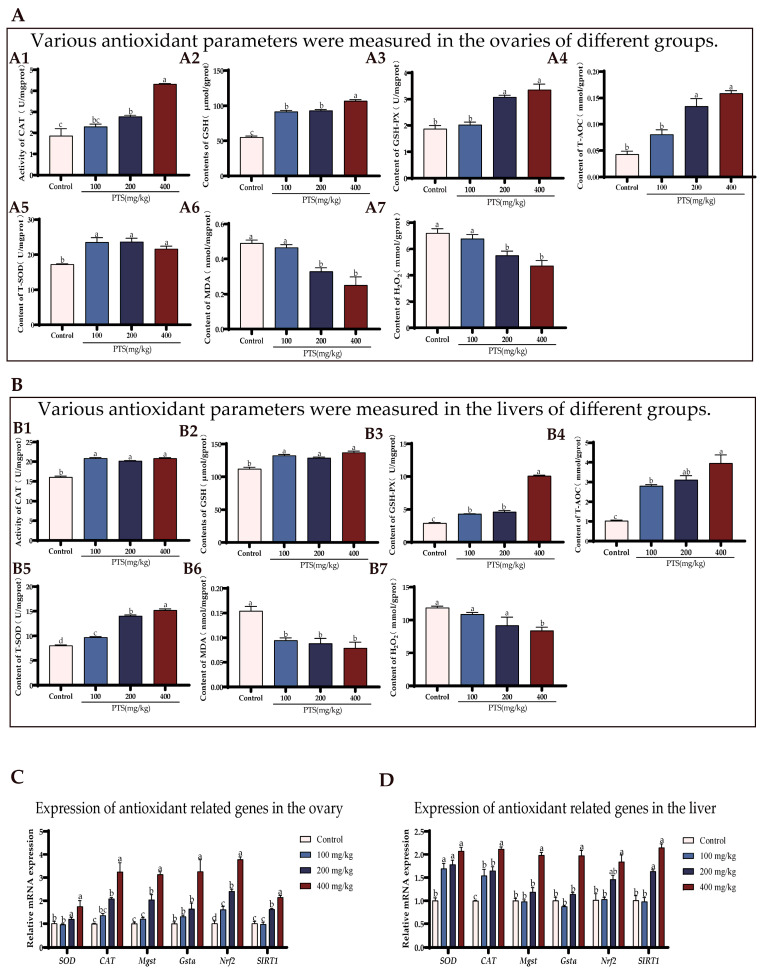
Effects of PTS supplementation on antioxidant parameters in ovaries and livers of D580 chickens. (**A**) Changes of various antioxidant enzymes measured in the ovaries of different groups.CAT (A1), GSH (A2), GSH-Px (A3), T-AOC (A4), T-SOD (A5), MDA (A6), H_2_O_2_ (A7). (**B**) Changes of various antioxidant enzymes measured in the livers of different groups. CAT (B1), GSH (B2), GSH-Px (B3), T-AOC (B4), T-SOD(B5), MDA(B6), H_2_O_2_ (B7). (**C**) Changes in genes related to antioxidant evaluated in the ovary of laying hens after feeding PTS. (**D**) Changes in genes related to antioxidant evaluated in the liver of laying hens after feeding PTS. All gene expression levels were analyzed with β-actin as a control. Significant differences between groups are shown by distinct lowercase letters in a test (*p* < 0.05).

To further explore the effect of PTS on laying hens, the antioxidant capacity of the ovaries and livers in each group was also examined. Figure 3 illustrates the various antioxidative stress biochemical indicators in the livers and ovaries of the four groups of laying hens. 

Supplementation with PTS increased CAT, GSH, GSH-Px, T-AOC, and T-SOD content in ovaries (Figure 3A(A1–A5)) and decreased MDA and H_2_O_2_ content (Figure 3A (A6, A7)). The results showed that PTS could improve ovarian antioxidant capacity in a dose-dependent manner. When the dietary PTS concentration reached 200 mg/kg and 400 mg/kg, the ovarian antioxidant capacity of laying hens was significantly higher compared to the control group.

In contrast to the control group, the liver samples from the group receiving PTS supplementation showed a notable enhancement in GSH, GSH-Px, T-SOD, T-AOC, and CAT levels. Conversely, there was a notable decrease in MDA and H_2_O_2_ content (Figure 3B(B1–B7)). Specifically, a significant decrease in ovarian H_2_O_2_ content was observed only when the PTS concentration reached 400 mg/kg (Figure 3B(B7)). 

The qRT-PCR results also demonstrated that feeding PTS increased the expression of antioxidation-related genes such as *CAT*, *SOD*, *Gsta*, and *Mgst* (Figure 3C, D). Interestingly, we also found that PTS supplementation promoted the expression of *SIRT1* and *Nrf2* genes in laying hens.

### 3.3. PTS Supplementation Demonstrates Anti-Apoptotic Effects on Ovary and Liver In Vivo

**Figure 4:**
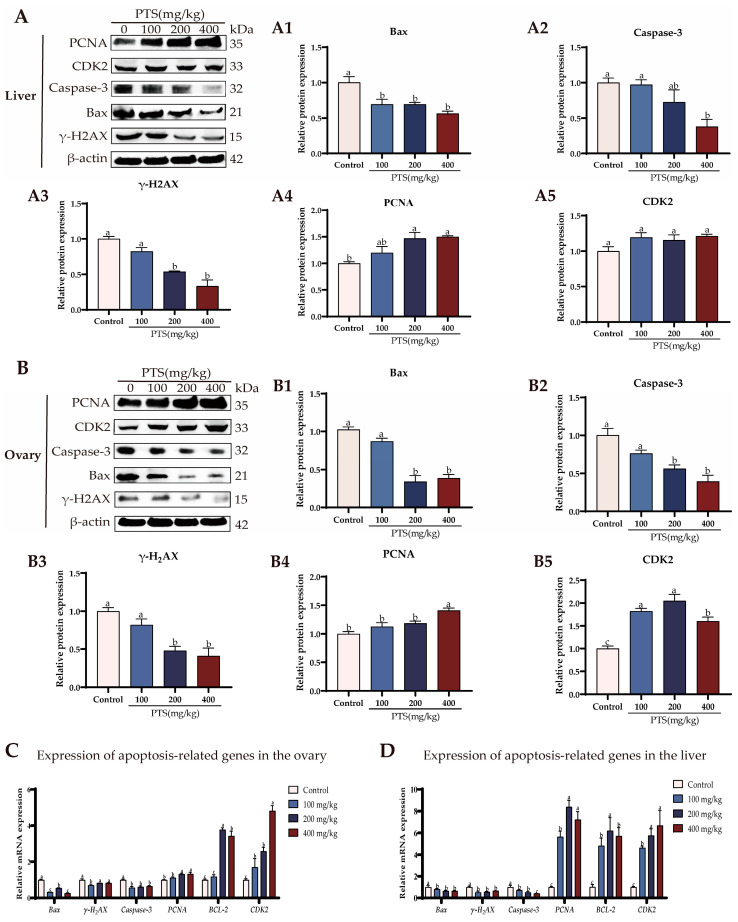
Effect of PTS supplementation on the anti-apoptosis ability of ovaries and livers of D580 chickens. (**A**) Changes in cell proliferation and apoptosis-related protein expression in the liver of laying hens after feeding PTS. Bax (A1), Caspase3 (A2), γ-H2AX (A3), PCNA (A4), CDK2 (A5). (**B**) Changes in cell proliferation and apoptosis-related protein expression in the ovary of laying hens after feeding PTS. Bax (B1), Caspase3 (B2), γ-H2AX (B3), PCNA (B4), CDK2 (B5). (**C**) Changes in cell proliferation and apoptosis-related gene expression in the ovary of laying hens after feeding PTS. (**D**) Changes in cell proliferation and apoptosis-related gene expression in the liver of laying hens after feeding PTS. All gene and protein expression levels were analyzed with β-actin as a control. Significant differences between groups are shown by distinct lowercase letters in a test (*p* < 0.05).

Supplementation with PTS reduced the expression of apoptosis-associated proteins, such as Bax, Caspase-3, and γ-H2AX (Figure 4A(A1–A3)), while increasing the expression of PCNA in livers compared to the control group (Figure 4A(A4)). Furthermore, there were no significant changes in CDK2 protein levels in livers following supplementation with PTS. (Figure 4A(A5)). 

Similar trends were observed in the ovaries, where PTS supplementation also led to decreased expression of Bax, Caspase3, and γ-H2AX (Figure 4B(B1–B3)) but increased the expression of PCNA (Figure 4B(B4)). However, different from the liver, there was a significant up-regulation of CDK2 protein expression in the ovary after feeding PTS (Figure 4B(B5)). 

The mRNA levels of the apoptosis gene were further estimated. As shown in Figure 4C,D, PTS supplementation significantly downregulated the expression level of *Bax* and Caspase-3, and increased expression levels of anti-apoptosisn related gene *BCL*-2, in both ovary and liver. The expression of proliferation—and cell cycle-related genes, *PCNA* and *CDK2*—also exhibited an increasing trend after feeding PTS compared to the control group (Figure 4C,D). 

**Figure 5:**
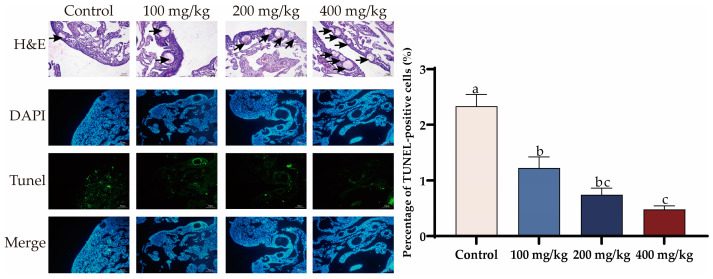
Effect of PTS supplementation on the Morphology changes in ovaries. Morphological effects of PTS on the ovary in vivo, as well as the effects of PTS on the TUNEL (green) index. Nuclei were stained with DAPI (blue). Scale bar: 50 μm. Follicles in the ovarian cortex (black arrow). Significant differences between groups are shown by distinct lowercase letters in a test (*p* < 0.05).

The effect of PTS on ovarian morphology in low-laying hens is shown in Figure 5 H&E staining revealed that feeding PTS increased the number of small follicles in the ovarian cortex of laying hens compared with the control group. Additionally, the rate of TUNEL-positive cells in the ovarian tissue was lower in the PTS supplement group than in the control group (Figure 5).

### 3.4. PTS Supplementation Demonstrates Fat Metabolism Effects on Ovary and Liver In Vivo

**Figure 6:**
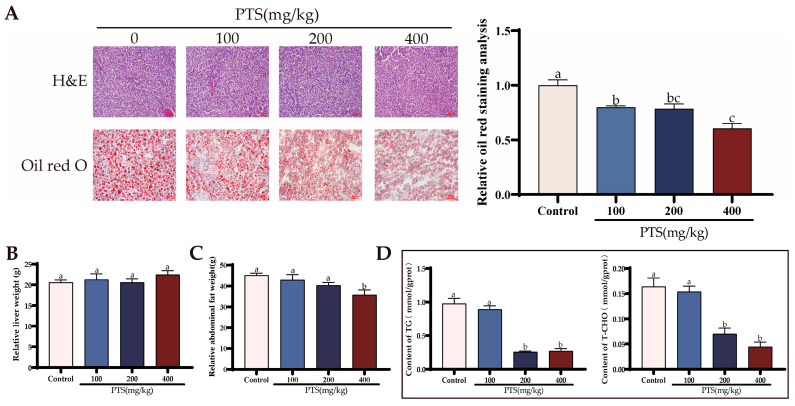
Effects of PTS supplementation on liver morphology, T-CHO and TG levels, and abdominal fat. (**A**) Morphological effects of PTS on the liver in vivo, as well as the Oil red staining of the liver. Scale bar: 50 μm and 20 μm. (**B**) The relative liver weight determined by calculating the liver weight to body weight ratio. (**C**) The relative abdominal fat weight assessed by calculating the abdominal fat weight to body weight ratio. (**D**) The content of total cholesterol (T-CHO) and triglycerides (TG) in livers. Significant differences between groups are shown by distinct lowercase letters in a test (*p* < 0.05).

To gain further insight into the potential effects of PTS on liver fat metabolism in laying hens, we conducted an investigation focusing on relevant indicators. Liver and abdominal fat were isolated and weighed separately in the four groups. PTS supplementation did not significantly affect liver weight across the different dosage groups (Figure 6B). However, a significant decrease in abdominal fat weight was observed at the highest dos-age of 400 mg/kg PTS (Figure 6C). These results suggest that PTS supplementation may specifically target and reduce abdominal fat in laying hens. 

Afterward, a morphological examination of the livers was then performed. H&E staining results indicated that the overall liver structure remained unchanged following PTS feeding (Figure 6A). However, oil red staining revealed a clear dose-dependent reduction in hepatic lipid droplets after 15 days of PTS administration. Notably, when PTS was ad-ministered at a dosage of 400 mg/kg, there was a remarkable 50.47% decrease in the relative area covered by lipid droplets in the liver of laying hens (Figure 6A).

We then measured the levels of TG and T-CHO in the liver. The results revealed a significant reduction in the levels of these two substances following PTS supplementation. Notably, the most prominent changes were observed when the PTS content in the diet reached 200 mg/kg and 400 mg/kg, suggesting a dose-dependent effect of PTS on decreasing TG and T-CHO levels in the liver (Figure 6D).

**Figure 7:**
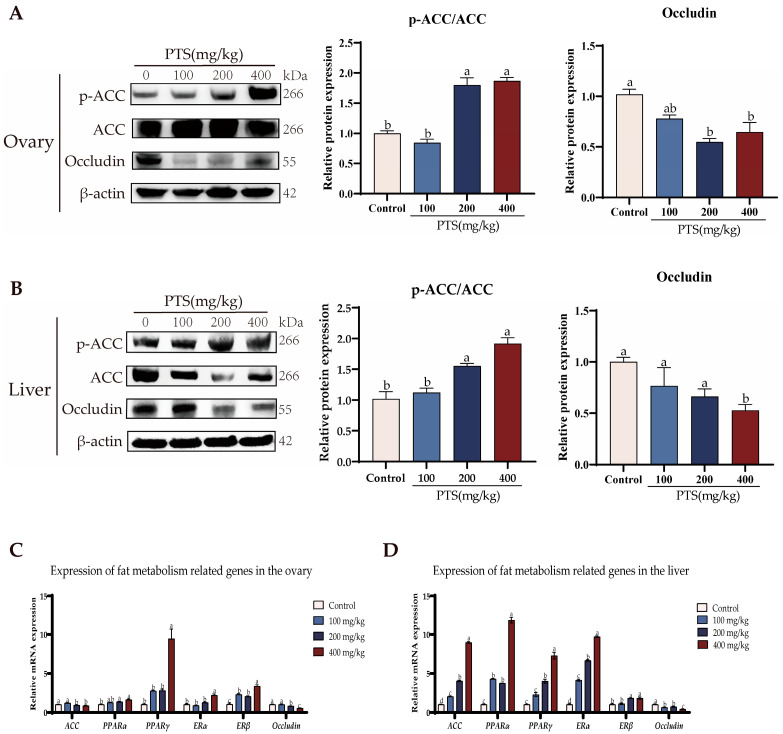
Effect of PTS supplementation on fat metabolism. (**A**) Relative protein expression of p-ACC, ACC and Occludin in ovary of laying hens after feeding PTS, with β-actin as the control. (**B**) Relative protein expression of p-ACC, ACC and Occludin in ovary of laying hens after feeding PTS (**C**) Relative expression of genes related to fat metabolism in the ovary. (**D**) Relative expression of genes related to fat metabolism in the liver. All gene and protein expression levels were analyzed with β-actin as a control. Significant differences between groups are shown by distinct lowercase letters in a test (*p* < 0.05).

Our investigation extended to analyzing the protein expression levels of ACC, p-ACC, and Occludin in liver and ovary tissues. Western blotting results demonstrated a significant up-regulation in the p-ACC/ACC ratio, indicating an activation of ACC phosphoryltion, and a noteworthy down-regulation in the expression of Occludin protein following PTS feeding in livers (Figure 7B). The trend of p-ACC/ACC protein expression in the ovary was consistent with that in the liver, but the protein expression of Occludin did not decrease with the increase of PTS concentration; instead, it increased at the dose of 400 mg/kg (Figure 7A).

Moreover, qRT-PCR analysis provided additional insights into gene expression changes in the livers and ovaries. It revealed that the expression of *PPARα*, *PPARγ*, *ERα*, and *ERβ* genes increased after PTS feeding, while the expression of the Occludin gene decreased compared to the control group (Figure 7C,D). Interestingly, following PTS feeding, *ACC* gene expression increased significantly in the liver, but decreased slightly in the ovary compared to the control group (Figure 7C,D).

These combined findings strongly suggest that PTS may stimulate fat metabolism in the liver, as evidenced by the increase in the expression of key genes involved in fat metabolism regulation. Additionally, the down-regulation of Occludin expression indicates that PTS may play a role in enhancing the transport of small molecules from the liver to the external environment.

### 3.5. PTS Exhibits Anti-Apoptotic and Anti-Oxidative Effects on D-gal-Induced Aged SWFs In Vitro

#### 3.5.1. PTS Exhibits Anti-Apoptotic Effects on D-gal-Induced Aged SWFs In Vitro

**Figure 8:**
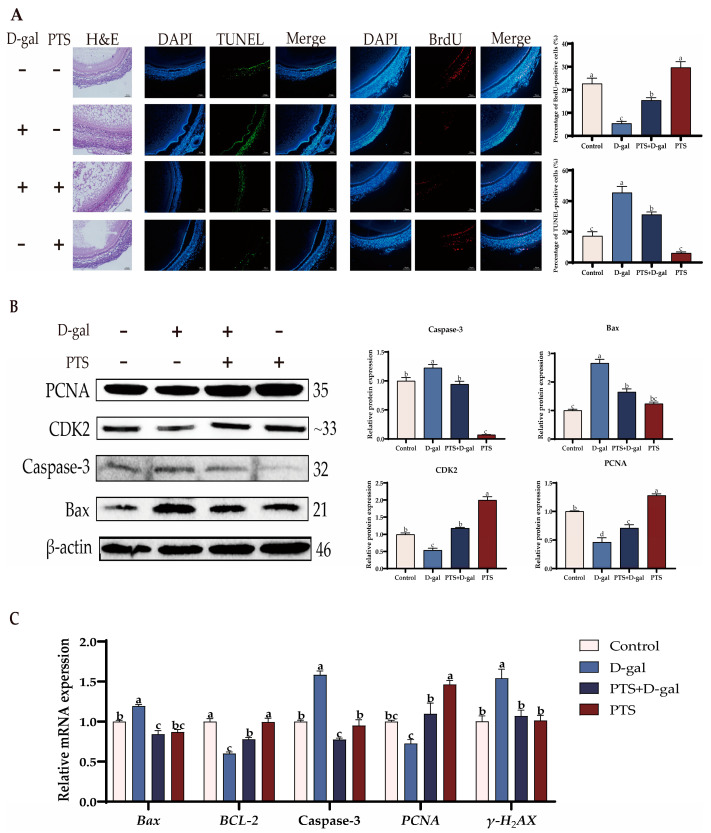
In vitro effects of PTS on D-gal-induced apoptosis in SWFs. (**A**) The effect of PTS on D-gal-induced morphological alterations in SWFs, as well as the effects of PTS on the BrdU (red) index and TUNEL (green) index induced by D-gal, were evaluated. The nuclei were stained with DAPI (blue). Scale bar: 50 μm. (**B**) Relative expression level of proteins involved in cell proliferation and apoptosis. (**C**) Relative expression level of genes involved in cell proliferation and apoptosis. All gene and protein expression levels were analyzed with β-actin as a control. Significant differences between groups are shown by distinct lowercase letters in a test (*p* < 0.05).

The SWFs were pretreated with a concentration of 0.5 μM PTS for 24 h, followed by treatment with 200 μM D-gal for 48 h to assess the impact of PTS on D-gal-induced oxidative stress injury. H&E staining demonstrated that the morphological alterations, characterized by the loosened shape of SWFs-GCs induced by D-gal, were effectively reversed after PTS pretreatment (Figure 8A). Notably, D-gal treatment alone led to a decrease in BrdU labeling rates, while the TUNEL assay showed a significantly higher percentage of TUNEL-positive cells in D-gal-induced SWFs compared to the control group (Figure 8A). 

Furthermore, Western blot analysis demonstrated that PTS pretreatment significantly alleviated D-gal-induced apoptosis in SWFs, as evidenced by reduced protein expression of Bax and Caspase-3. Interestingly, PTS pretreatment also induced the expression of proliferation-related proteins, such as PCNA and CDK2, in SWFs (Figure 8B). Additionally, qRT-PCR results indicated a substantial decrease in mRNA levels of *Bcl-2* and *PCNA* after D-gal treatment, whereas *Bax*, Caspase-3, and *γ-H2AX* were significantly up-regulated. Pretreatment with PTS for 24 h effectively revived the gene expression to match the levels observed in the control group (Figure 8C).

#### 3.5.2. PTS Exhibits Anti-Oxidative Stress Effects on D-gal-Induced Aged SWFs In Vitro

**Figure 9:**
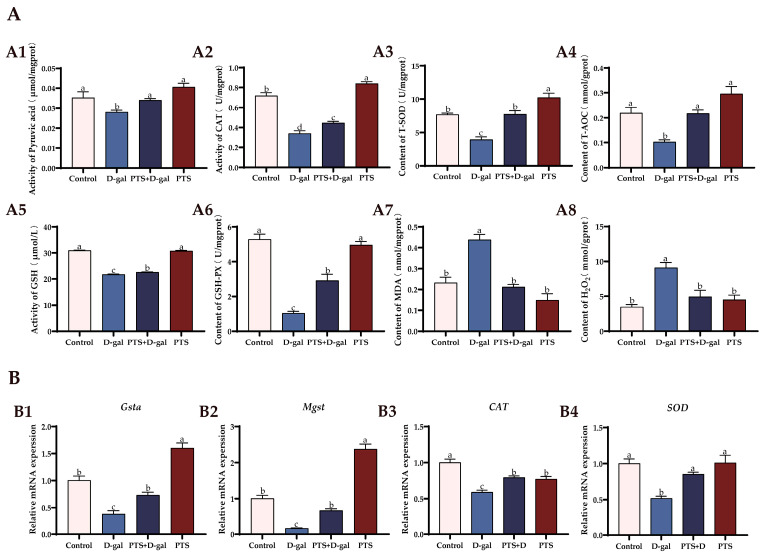
In vitro antioxidant effects of PTS on D-gal-induced oxidative stress in SWFs. (**A**) The antioxidant enzyme levels of pyruvic acid (A1), CAT (A2), T-SOD (A3), T-AOC (A4), GSH (A5), GSH-Px (A6) as well as the levels of oxidation products MDA (A7) and H2O2 (A8). (**B**) Relative expression of genes related to antioxidant, with β-actin as the control. *Gsta* (B1), *Mgst* (B2), *CAT* (B3), *SOD* (B4). Significant differences between groups are shown by distinct lowercase letters in a test (*p* < 0.05).

The antioxidant capacity of SWFs was compared among the different groups. The levels of T-AOC, T-SOD, CAT, GSH, GSH-Px, and Pyruvic acid in SWFs were significantly reduced after treatment with D-gal alone, while the content of MDA and H_2_O_2_ was increased (Figure 9A(A1–A8)). However, pretreatment with PTS prevented these trends, enhanced the antioxidant capacity of SWFs, and protected SWFs from oxidative stress injury. The expression levels of *Cat, SOD, Mgst,* and *Gsta* mRNAs were significantly reduced by D-gal treatment compared with the control group, as revealed by the qRT-PCR results. However, PTS pretreatment standardized these changes (Figure 9B(B1–B4)). These findings suggest that PTS restored the declining antioxidant capacity and rescued the altered expression of antioxidant genes in aging SWFs induced by D-gal.

### 3.6. PTS Protected D-gal-Induced Aged SWFs from Oxidative Stress and Apoptosis via Increased SIRT1/Nrf2 Expression

**Figure 10:**
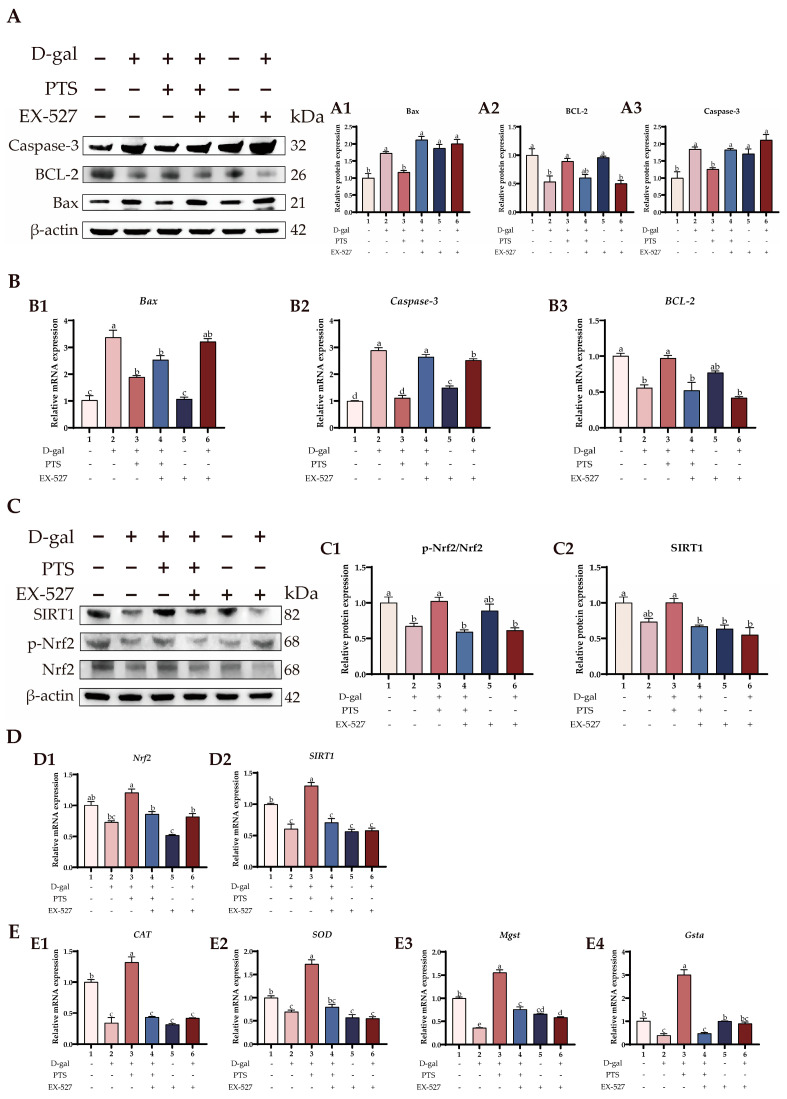
The suppression of EX-527 on the promotion of cell antioxidant ability and inhibition of cell apoptosis by PTS. (**A**) Effect of different treatments on the expression of apoptosis-related protein expression. Bax (A1) BCL-2 (A2), Caspase3 (A3). (**B**) Effect of different treatments on the expression of apoptosis-related genes expression. Bax (B1) Caspase3 (B2), BCL-2 (B3). (**C**) Effect of different treatments on the expression of Nrf2, p-Nrf2 and SIRT1 protein expression. p-Nrf2/Nrf2 (C1), SIRT1 (C2). (**D**) Effect of different treatments on the expression of Nrf2 and SIRT1 genes expression. *Nrf2* (D1), *SIRT1* (D2). (**E**) Effect of different treatments on the expression of antioxidant-related genes expression. *CAT* (E1), *SOD* (E2), *Mgst* (E3), *Gsta* (E4). All gene and protein expression levels were analyzed with β-actin as a control. Significant differences between groups are shown by distinct lowercase letters in a test (*p* < 0.05).

Figure 10A(A1–A3) demonstrates that PTS treatment reduced the protein levels of Bax and Caspase-3 while inducing the expression of BCL-2 protein compared with the D-gal-induced group. However, the anti-apoptotic effect of PTS was greatly weakened after pre-treatment with EX-527. Furthermore, the qRT-PCR analysis revealed that the inhibitor EX-527, countered the downregulation of apoptosis-related genes induced by PTS. Specifically, in comparison to the D-gal-induced group, the PTS + D-gal group showed a notable rise in the levels of *BCL-2* mRNAs, whereas the expression of *Bax* and Caspase-3 significantly dropped (Figure 10B(B1–B3)).

Further experiments found that PTS administration increased the significantly reduced p-Nrf2/Nrf2 ratio in the D-gal-induced SWFs (Figure 10C(C1)). However, pretreatment with EX-527 significantly blocked PTS-mediated phosphorylation of Nrf2. Similarly, upstream protein SIRT1 levels were enhanced in response to PTS treatment. However, pretreatment with EX-527 markedly counteracted the increase in SIRT1 levels induced by PTS (Figure 10C(C2)). These findings strongly suggest that PTS treatment alleviates oxidative stress and ameliorates apoptosis in the D-gal-induced aging SWFs via the SIRT1/Nrf2 pathway. Similarly, EX-527 pretreatment decreased the expression of *SIRT1* and *Nrf2* mRNAs in SWFs. In contrast, PTS pretreatment of SWFs significantly countered the decrease in *SIRT1* and *Nrf2* mRNA expression caused by D-gal, thereby restoring the antioxidant capacity of SWFs. However, the addition of EX-527 offset the PTS-induced promotion of *SIRT1* and *Nrf*2 mRNAs expression (Figure 10D (D1, D2)).

Finally, we examined the antioxidant genes involved. The results indicate that PTS pretreatment can mitigate the down-regulation of *CAT*, *SOD*, *Mgst*, and *Gsta* genes induced by D-gal, but this mitigating effect can be inhibited by EX-527 (Figure 10E (E1–E4)).

## 4. Discussion

Laying hens typically reach a peak laying period around 280 days of age, with a de-cline in both laying rate and breeding value becoming noticeable around 580 days of age, marking the onset of senescence. Therefore, we designated 280 days of age as the period of high-yield laying hens and 580 days of age as the stage of senescent hens. As animals age, various organs undergo gradual senescence, accompanied by intracellular accumulation of ROS. Oxidative stress arises from an imbalance between the antioxidant system and the production of oxidants. This imbalance can lead to a range of diseases, such as cancer, cardiovascular disease, and inflammatory conditions [35]. These pathologies often involves oxidative modifications of key physiological molecules such as proteins, lipids, carbohydrates, and nucleic acids, as well as dysregulation of gene expression and inflammatory responses [36]. Due to the laying performance of hens, there is a strong demand for lipid metabolism and oxidation in their bodies, which leads to excessive lipid accumulation in the later stages of laying, thereby reducing production performance [37]. Mitigating oxidative stress generation proves to be an effective strategy for preventing various diseases and extending animal lifespan [38]. Among the organs affected by aging and oxidative stress, the ovary is particularly vulnerable [13,39]. The accumulation of oxidative stress disrupts the balance between ovarian cell proliferation and apoptosis, resulting in an increase in atretic follicles and a decline in ovarian function [40,41].

An increasing body of evidence supports the supplementation of antioxidants as an effective strategy for reducing oxidative stress in animals [42]. PTS, a derivative of resveratrol, is predominantly found in mulberries, grapes, blueberries, and other berries. PTS has been extensively studied for its potential therapeutic effects in diseases such as diabetes, inflammation, and cancer [24,43]. While PTS shares similarities with resveratrol, it exhibits superior clinical efficacy due to slight structural differences. Specifically, PTS demonstrates stronger intestinal absorption capacity, liver stability, and bioavailability compared to resveratrol, along with lower biotoxicity [44,45]. Dietary supplementation of PTS can improve the immune and antioxidant function and intestinal health of broilers [46]. In this study, we investigated the antioxidant and anti-apoptotic effects of PTS on D-gal-induced SWFs and naturally aged laying hen SWFs. Additionally, through a feeding experiment, we explored the impact of PTS supplementation on the overall health and physiological conditions of laying hens.

Given that the accelerated aging process in the D-gal-induced aging model closely mirrors human aging, this model has become a cornerstone for investigating the mechanisms underlying ovarian aging [28]. Generally, apoptosis serves as a mechanism for cell renewal and tissue stability [47]. However, excessive tissue apoptosis can lead to organ dysfunction, disrupting normal physiological activities. In our prior laboratory investigations, we observed that D-gal induces senescence in GCs of laying hens, evidenced by an increase in the positive rate of GCs SA-β-gal [48]. In this study, we directly treated SWFs of D280 laying hens with D-gal. An increase was observed in MDA and H_2_O_2_ content, coupled with a decrease in various antioxidant indexes, confirming the induction of oxidative stress. Pretreatment with PTS effectively mitigated the decline in antioxidant capacity induced by D-gal, primarily by upregulating the expression of antioxidants such as GSH, GSH-Px, CAT, T-SOD, and T-AOC. Knockdown of antioxidant genes has been shown to reduce the proliferation of human granular cells and disrupt intracellular ROS homeostasis [41]. Our research revealed that PTS pretreatment increased the expression of antioxidation-related genes, including *CAT*, *SOD*, *Mgst*, and *Gsta*, in both D-gal-induced and naturally senescent SWFs. Elucidating the mechanisms underlying ovarian aging and attenuating oxidative stress may extend the ovarian lifespan of laying hens, thereby enhancing laying performance and reducing industry losses.

During aging, there is an increase in DNA damage (as indicated by γ-H2AX positive cells) and apoptosis rate (TUNEL-positive cells) in ovarian GCs [41]. Our experimental results showed that PTS could effectively reduce the positive rate of TUNEL and the expression of γ-H2AX in cells. P53 responds to various types of cellular stress, such as DNA damage, hypoxia, and oxidative stress [49]. Relevant research shows that P53 plays a key role in apoptosis induced by DNA damage, and inhibition of P53 expression can promote the expression of CDK2 protein [50]. Our study found that PTS can increase the expression of the CDK2 protein in the ovaries of laying hens both in vivo and in vitro, with a greater effect observed in the ovaries compared to the liver. However, whether PTS alleviates apoptosis through its interaction with P53 still needs to be further studied.

Apoptosis induced by ROS accumulation primarily occurs through internal pathways rather than external ones [51]. Endogenous apoptosis is regulated by the B cell lymphoma 2 (BCL-2) family, where the BH3-only protein detects the apoptotic signals and transmits them to other BCL-2 family members, ultimately initiating the apoptotic cascade. This process leads to the activation of Bax, BAK, and BOK, which bind and inhibit core anti-apoptotic BCL-2 proteins [52]. Caspase-3, a frequently activated death protease, catalyzes the specific cleavage of many key cell proteins and can be activated either after the formation of an apoptotic complex composed of Cytc and Caspase-9 or directly [53]. In this study, we observed upregulation of the protein and mRNA expressions of Bax, Caspase-3, and γ-H2AX in SWFs following D-gal treatment. Conversely, the expression levels of genes and proteins related to cell cycle and proliferation, such as CDK2 and PCNA, were downregulated, along with a similar trend observed in the expression levels of BCL-2 genes and proteins. PTS treatment reversed these D-gal-induced changes, indicating that PTS regulates the homeostasis of SWFs’ cell proliferation and apoptosis. Notably, a similar effect was observed in D580 chickens fed PTS, where the apoptosis of ovarian cells in laying hens was significantly alleviated after PTS feeding.

In poultry, the liver, ovary, and blood are crucial for regulating egg production [54]. Thus, lipid metabolism in both the ovaries and liver plays a significant role in subsequent yolk deposition, egg quality and yield. Our study revealed that feeding PTS significantly increased the number of SYFs and SWFs in the ovaries of laying hens, with SYFs showing a notable 34.7% increase compared to the control group. Furthermore, PTS decreased the levels of TG and T-CHO in the liver and blood of low-laying hens, reduced the size of lipid droplets in the liver, and lowered the relative weight of abdominal fat. These findings collectively suggest that PTS positively influences fat metabolism in laying hens.

During egg yolk deposition, carbohydrates are converted into triglycerides or cholesterol esters by key enzymes, such as ACC and fatty acid synthetase (FASN) [55,56]. Lipids are essential for the production of anabolic steroid hormones in GCs, and hepatic oxidative stress can disrupt hepatic lipid metabolism [57]. Studies have shown that oxidative stress caused by direct H_2_O_2_ treatment on SWFs can decrease the expression levels of PPARγ, VLDLR, ER-α, and other genes related to fat synthesis and transport [58]. ACC plays a crucial role in controlling the metabolism of carbohydrates and fatty acids. The reduction of ACC phosphorylation has been associated with the development of hepatocellular carcinoma in mice and increased the proliferation of human liver cancer cells [59]. Studies indicates that the fat content in the liver of aging or low-producing hens is significantly higher than that of high-producing hens, and the expression levels of lipolysis and transport-related genes such as *ACC*, *PPARα*, and *PPARγ* decrease significantly with aging [58,60,61]. Our experiments demonstrated that dietary supplementation with PTS promoted the phosphorylation of ACC protein in the liver and ovary, and significantly increased the gene expression of PPARα and PPARγ in both organs of laying hens.

Glutathione-S-transferase (GST) is a group of enzymes associated with liver detoxification, comprising families such as GSTA, GSTK, GSTO, GSTP, GSTT, GSTZ, and MGST [62]. Among these, the genes *Mgst* and *Gsta* are known to be associated with antioxidative properties [63], the enzymes they encode assist cells in combating oxidative stress by eliminating free radicals and other harmful oxidants, thereby protecting cells from oxidative damage. Our research revealed that supplementing PTS to laying hens led to higher levels of antioxidants, such as GSH, GSH-Px, T-AOC, T-SOD, and CAT in both the liver and ovary. Additionally, PTS supplementation decreased markers of oxidative stress, including MDA and H_2_O_2_, and promoted the expression of antioxidant-related genes such as *Mgst*, *SOD*, *Gsta*, and *CAT*. These results indicate that incorporating PTS into the diet can enhance the antioxidant capabilities of laying hens.

Sirtuins are pivotal in regulating metabolism, cell proliferation, and genome stability [14]. Among the Sirtuins family, SIRT1, SIRT3, and SIRT6 are considered potential markers of ovarian aging, with SIRT1 notably showing reduced expression in oxidative stress-induced premature aging of the ovaries (POI) [14,64]. Our study found a significant decrease in SIRT1 and Nrf2 expression levels in SWFs after D-gal treatment, which was reversed by pretreatment with PTS. However, this beneficial effect of PTS was markedly inhibited by pretreatment with EX-527, a SIRT1 inhibitor, suggesting that PTS influences SWFs via the SIRT1 pathway. Additionally, the Sirtuins are believed to reduce the accumulation of ROS by deacetylating Nrf2 and promoting the production of antioxidant enzymes [17]. Numerous experiments have demonstrated that natural plant extracts can exert antioxidative and anti-apoptotic effects by activating the SIRT1/Nrf2 pathway [18,65,66]. Thus, whether PTS can alleviate oxidative stress-induced damage in the aging ovary through the SIRT1/Nrf2 pathway remains to be explored. Our results indicate that PTS pretreatment can counteract the decrease in *CAT*, *SOD*, *Mgst*, and *Gsta* mRNA expression caused by D-gal, an effect impeded by EX-527. Similarly, we observed that PTS promotes BCL-2 expression and inhibits Bax and Caspase-3 expression through the SIRT1/Nrf2 pathway, thereby alleviating apoptosis in D-gal-induced SWFs.

## 5. Conclusions

**Figure 11:**
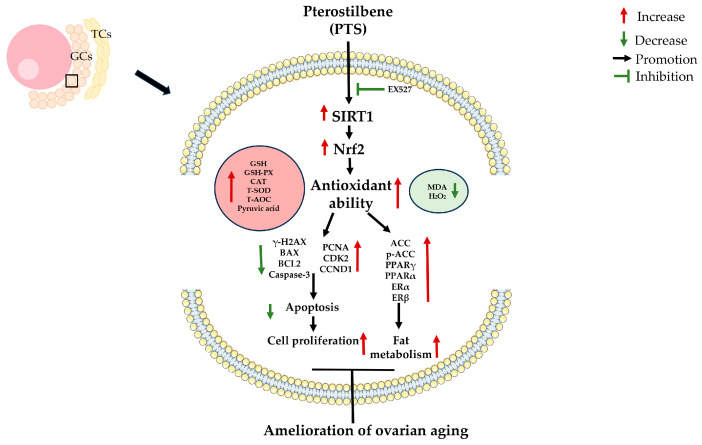
Antioxidant effect of PTS on ovaries in the aging chickens.

This study highlights the efficacy of PTS in alleviating oxidative stress both in vitro and in vivo, thereby mitigating adverse effects such as inhibited cell proliferation and promoted cell apoptosis. PTS exerts its protective effects by activating the SIRT1/Nrf2 pathway, which enhances antioxidant capacity, prevents follicular atresia and maintains ovarian quality. While the study demonstrates that PTS can alleviate oxidative stress and reduce apoptosis, the precise mechanisms through which apoptosis is mitigated require further investigation. These findings suggest that PTS holds potential as a feed supplement in poultry production to improve ovarian health and performance (Figure 11).

## Figures and Tables

**Table 1:** Primers sequences for PCR analysis.

Genes	Accession No.	Primer Sequence (5′–3′)	Product Size (bp)
*CDK-2*	NM_001199857.1	F: TCCGTATCTTCCGCACGTTG R: GCTTGTTGGGATCGTAGTGC	183
*PCNA*	NM_204170.2	F: GGGCGTCAACCTAAACAGCA R: AGCCAACGTATCCGCATTGT	97
Caspase-3	NM_204725.1	F: CAGCTGAAGGCTCCTGGTTT R: GCCACTCTGCGATTTACACG	98
*BCL-2*	NM_205339.2	F: ATCGTCGCCTTCTTCGAGTT R: ATCCCATCCTCCGTTGTCCT	150
*Bax*	XM_015290060.2	F: GGATGACAGGAAAGTACGGCA R: TCACCAGGAAGACAGCGTAT	173
*γ-H2AX*	BM488821.1	F: AACAAGAAGACGCGCATCAT R: GTAGCACGGCCTGAATGTT	143
*Mgst*	NM_001135550.1	F: GGCATTTGCCAACCCAGAAG R: CAAGGTCATTCAGGTGGCCT	116
*Gsta*	NM_204818.2	F: GCAGAGCCATCCTCAGCTAC R: CCTTTGCCTCAGGTGGAGAG	150
*Cat*	NM_001031215.2	F: TCAGGAGATGTGCAGCGTTT R: TCTTACACAGCCTTTGGCGT	109
*SOD*	NM_205064.1	F: GGCAATGTGACTGCAAAGGG R: CCCCTCTACCCAGGTCATCA	133
*Nrf2*	NM_001030756.1	F: CTGCTAGTGGATGGCGAGAC R: CTCCGAGTTCTCCCCGAAAG	132
*SIRT1*	NM_001004767.2	F: CCCCGCAGCCCGATAAC R: ATACGTGGTCTTGGGGTCCA	127
*ERα*	NM_205183.2	F: CAGGCCTGCCGACTAAGAAA R: TTCATCATTTCGCCGCCTCT	86
*ERβ*	NM_204794.2	F: ACCCCATTCAGTGTCAATCAGG R: AGCCAATACCAGCAGGTGAG	163
*ACC*	NM_205505.1	F: GTCTGCTCAACTGCGACCG R: CAAAGCGACTTCCTCTGGTCAGT	102
*PPARα*	NM_001001464.1	F: TAGTAAGCTCTCAGAAACTTT R: GAAACAGAAGCCGCTTTCCA	157
*PPARγ*	NM_001001460.1	F: GCTGTGAAGTTCAACGCACT R: CCTGGGCGATCTCCACTTAG	87
Occludin	NM_205128.1	F: CTCTGGGAAGGGCTGAGGT R: GCCTTCCCAAAAAGCCCTGA	170
β-actin	NM_205518	F: ACACCCACACCCCTGTGATGAA R: TGCTGCTGACACCTTCACCATT	136

**Table 2:** Effects of PTS supplementation on physical quality of eggs.

Item	Control	100 mg/kg	200 mg/kg	400 mg/kg	*p*-Value
Egg weight (g)	59.30 ± 0.493	61.38 ± 2.311	58.98 ± 1.984	59.85 ± 1.154	0.742
Shell strength (ksf)	3.75 ± 0.169	3.50 ± 0.166	4.09 ± 0.303	3.54 ± 0.160	0.204
Haugh Unit	76.77 ± 6.250 ^b^	83.15 ± 4.204 ^ab^	92.72 ± 1.882 ^ab^	93.82 ± 2.814 ^a^	0.024
Shell thickness (mm)	0.37 ± 0.011	0.37 ± 0.008	0.37 ± 0.009	0.36 ± 0.011	0.561
Egg yolk index	5.97 ± 0.170 ^b^	6.58 ± 0.128 ^a^	6.62 ± 0.120 ^a^	6.58 ± 0.178 ^a^	0.017

^a,b^ Means within a row with different superscripts differ significantly (*p* < 0.05).

**Table 3:** Effects of PTS supplementation on serum biochemical parameters.

Item	Control	100 mg/kg	200 mg/kg	400 mg/kg	*p*-Value
ALT	10.60 ± 1.430	10.89 ± 1.376	10.11 ± 0.695	7.87 ± 0.476	0.213
AST	269.50 ± 3.863 ^a^	266.00 ± 5.092 ^a^	259.70 ± 3.058 ^a^	237.20 ± 6.501 ^b^	0.0005
GLU	12.05 ± 0.090	12.26 ± 0.170	12.26 ± 0.169	11.91 ± 0.353	0.618
HDL	0.77 ± 0.147	0.71 ± 0.078	0.66 ± 0.053	0.52 ± 0.033	0.252
LDL	2.14 ± 0.237 ^a^	1.76 ± 0.212 ^ab^	1.63 ± 0.190 ^ab^	1.29 ± 0.079 ^b^	0.032
TC	10.08 ± 0.126 ^a^	5.37 ± 0.631 ^b^	5.33 ± 0.652 ^b^	3.84 ± 0.170 ^b^	<0.0001
TG	24.06 ± 0.607 ^a^	22.50 ± 0.179 ^b^	22.53 ± 0.304 ^b^	19.10 ± 0.327 ^c^	<0.0001

^a,b,c^ Means within a row with different superscripts differ significantly (*p* < 0.05).

## Data Availability

All data analyzed are contained within the article.
